# Structure–Activity Relationship and Stability Mechanism of Pickering Emulsions Stabilized by Gorgon Euryale Starch–Quinoa Protein Complex Under pH Regulation

**DOI:** 10.3390/foods15020211

**Published:** 2026-01-07

**Authors:** Xuran Cai, Guilan Zhu, Xianfeng Du

**Affiliations:** 1 School of Biology and Food Engineering, Hefei Normal University, Hefei 230601, China; 2 Anhui Engineering Laboratory for Agro-Products Processing, Anhui Agricultural University, Hefei 230036, China

**Keywords:** pH, gorgon euryale starch, quinoa protein, interfacial structure, Pickering emulsion

## Abstract

This study investigated the effects of pH (3, 5, 7, 9, 11) on the structure–activity relationship and stability mechanism of Pickering emulsions stabilized by the gorgon euryale starch–quinoa protein complex. Analyses were performed using reverse compression test, rheology, thermal stability assessment, atomic force microscopy (AFM), and low-field nuclear magnetic resonance (LF-NMR) measurements. Reverse compression test showed that the emulsion at pH 3 exhibited the highest hardness and consistency, but the weakest cohesiveness. Rheological measurements revealed that all emulsions displayed shear-thinning behavior, the emulsion at pH 3 had the highest shear stress and apparent viscosity, while that at pH 11 showed the lowest viscosity due to the destruction of macromolecular structures. Thermal stability assessment indicated that the emulsion at pH 3 did not undergo significant stratification even at 60 °C, whereas the stability of emulsions decreased between pH 5–9. Microscopic analyses (optical microscopy, AFM, and LF-NMR) further confirmed that the emulsion at pH 3 had fine, uniform droplets, strong water-binding capacity, and an interfacial film with a “dense protrusion” structure. This study provides a basis for the environmental adaptability design of functional emulsions and contributes to the high-value utilization of gorgon euryale and quinoa resources.

## 1. Introduction

Pickering emulsions, as a dispersion system stabilized by solid particles instead of traditional surfactants, exhibit great application potential in food, medicine, cosmetics, and other fields due to their excellent interfacial stability, biocompatibility, and controllability [1]. Natural biological macromolecules have emerged as ideal candidates for preparing Pickering stabilizers owing to their wide availability, high safety, and diverse functionalities [2]. Among them, starch–protein composite particles can significantly enhance emulsion stability and functionality through synergistic effects, making them a research focus in recent years [3].

Most conventional starches (e.g., corn starch, potato starch) have large particle sizes and weak interfacial adsorption capacity. When used alone as Pickering stabilizers, they are difficult to form dense and continuous interfacial films at the oil–water interface, which easily leads to system flocculation and failure to effectively resist droplet coalescence and stratification [4]. The interfacial activity of conventional plant proteins (e.g., soybean protein, wheat protein) is susceptible to pH changes; they tend to aggregate and destabilize near the isoelectric point. Additionally, the interfacial films formed by their sole adsorption have insufficient mechanical strength, making them hard to withstand processing stresses such as heat and shear [5].

Gorgon euryale starch (GES) is derived from the seeds of gorgon euryale, which is widely cultivated in Anhui, Jiangsu, and other regions of China [6]. Characterized by small particle size, good film-forming properties, and potential interfacial adsorption capacity, gorgon euryale starch can form a three-dimensional network through hydration in food systems, thereby enhancing system stability [7]. Quinoa protein (QP), extracted from quinoa seeds originating from the Andean region of South America, is a high-quality plant protein that contains all essential amino acids for humans, with high digestibility and low allergenicity. It can exhibit excellent interfacial activity through conformational changes under different environments, effectively adsorbing at the oil–water interface and reducing interfacial tension [8]. In our previous studies, it has been found that GES and QP can form composite particles through hydrogen bonding and hydrophobic interactions. The resulting synergistic effect significantly enhances the mechanical strength of the interfacial film, enabling the stabilization of Pickering emulsions by regulating interfacial behavior, which provides a certain basis for the development of natural composite stabilizers [9]. As characteristic agricultural products, the deep processing and utilization value of gorgon euryale and quinoa have not been fully explored. Developing GES and QP as high-value Pickering emulsion stabilizers not only improves the added value of agricultural products but also endows emulsions with natural and safe properties, which meets the market demand for “clean label” products in food, cosmetics and other fields. Compared with conventional raw materials, the GES-QP composite system not only ensures emulsion stability but also introduces nutritional functionality, expanding the application scenarios and market competitiveness of products.

However, the stability of Pickering emulsion systems is often significantly affected by environmental factors, among which pH, as a key parameter in food processing and storage, has a particularly prominent impact on emulsions [10]. Studies have shown that pH can directly affect the interfacial activity of composite particles and the macroscopic properties of emulsions by altering the charge state, molecular conformation, and interaction modes of biological macromolecules [11]. For example, when the system pH is far from the isoelectric point of proteins, the net charge on protein particles increases, leading to enhanced electrostatic repulsion between droplets, which can effectively prevent droplet aggregation [12]. For polysaccharide–protein composite systems, pH changes may affect hydrogen bonding, electrostatic, and hydrophobic interactions between the two components, thereby altering the adsorption behavior of composite particles at the oil–water interface and the structure of the interfacial film. Currently, the mechanism by which pH regulates the microstructure and functional properties of the GES-QP complex to affect the stability of Pickering emulsions remains unclear, which limits the precise application of this composite system in complex food environments.

Based on this, this study focuses on the structure–activity relationship and stability mechanism of Pickering emulsions stabilized by the GES-QP complex under pH regulation. By systematically investigating the effects of different pH conditions (3, 5, 7, 9, 11) on the texture, rheological behavior of Pickering emulsions, combined with microscopic morphology observation and stability evaluation, this study aims to reveal the intrinsic law by which pH affects emulsion stability through regulating the “structure–function” correlation of the complex. This research provides an experimental basis for designing functional emulsions with strong environmental adaptability and opens up new avenues for the high-value utilization of gorgon euryale and quinoa resources.

## 2. Materials and Methods

### 2.1. Materials

Gorgon euryale whole powder (food grade, moisture content: 9.41%, *w*/*w*) was supplied by Xi’an Tuofeng Biotechnology Co., Ltd. (Xi’an, China). QP (food grade, purity > 99%, moisture content: 9.82%, *w*/*w*) was provided by Shanxi Tianzhicheng Biotechnology Co., Ltd. (Xi’an, China). Peanut oil was obtained from Sanhe Huifu Grain and Oil Group Co., Ltd. (Sanhe, China). All other chemicals and reagents were of analytical grade and purchased from Sinopharm Chemical Reagent Co., Ltd. (Shanghai, China).

### 2.2. Extraction and Preparation of GES

The extraction and preparation of GES were conducted in accordance with the method previously explored by us [9]. To better retain the key functional properties of GES, such as its inherent small particle size and film-forming ability, a mild alkaline extraction method was employed in this study. First, 200 mL of 0.3% sodium hydroxide solution was prepared in a beaker. Then, 80 g of gorgon euryale whole powder was weighed and added into the sodium hydroxide solution, followed by continuous stirring to form a uniform suspension of gorgon euryale whole powder. The suspension was placed at room temperature for 1 h, with stirring at a rate of 200 r/min during this period. After 1 h, the upper alkali solution was decanted, and the remaining suspension of gorgon euryale whole powder was transferred into a Buchner funnel for suction filtration. The solid material was retained and repeatedly washed with distilled water until neutral during the process. Subsequently, the solid material was dried in a constant-temperature drying oven at 45 °C for 24 h. After drying, it was initially crushed and then ball-milled in a ball mill (BXQM-2L, Nanjing Telenew Instrument Co., Ltd., Nanjing, China) at a rotation speed of 280 r/min for 2 h to obtain the desired GES.

### 2.3. Preparation of GES-QP Complex Pickering Emulsions Under Different pH Conditions

At room temperature, 0.05 mol/L buffer solutions were prepared separately: citric acid–sodium citrate buffer for pH 3 and 5, sodium dihydrogen phosphate–disodium hydrogen phosphate buffer for pH 7, and sodium carbonate–sodium bicarbonate buffer for pH 9 and 11 (all adjusted to the target pH with citric acid or sodium hydroxide for fine-tuning). Five beakers were taken, and 50 mL of buffer solution corresponding to each pH (3, 5, 7, 9, and 11) was accurately measured into each beaker. Then, GES and QP were added into each beaker at a mass ratio of 8:2, resulting in a total solid content of 10% (*w*/*w*). This 8:2 mass ratio was selected based on the conclusions of our previously published study [9], where systematic screening of GES-QP ratios (10:0, 9:1, 8:2, 7:3, 6:4, 5:5) demonstrated that this ratio maximized the synergistic interaction between GES and QP-GES provided a stable structural framework via its excellent film-forming property, while QP enhanced interfacial adsorption, leading to the highest emulsification stability. The specific order of adding the complex components was as follows: GES was first added into the buffer solution and stirred until uniform, and then QP was added and stirred until uniform. Subsequently, soybean oil with a volume fraction of 20% was added into the beaker, and emulsification was performed using an emulsifier (T18 brushless digital, IKA, Staufen im Breisgau, Germany) at a rotation speed of 22,000 r/min for 2 min. Finally, GES-QP complex Pickering emulsions under different pH conditions were obtained, which were named as pH 3, pH 5, pH 7, pH 9, and pH 11, respectively.

### 2.4. Reverse Compression Test

The determination was performed according to the method described by Liu et al. with appropriate modifications [13]. An appropriate amount of freshly prepared emulsion sample was placed in a beaker, and the reverse compression test was conducted using a texture analyzer (TA-XT2i, Stable Micro Systems, Godalming, UK). The test mode was set to compression with a P/36R probe. The pre-test speed, test speed, and post-test speed were 1 mm/s, 1 mm/s, and 10 mm/s, respectively. The target mode was set to distance with a value of 10 mm, and the trigger force was 2 g. Characteristic parameters including Firmness, Consistency, Cohesiveness, and Work of Cohesion were recorded.

### 2.5. Determination of Static Rheological Properties

An appropriate amount of freshly prepared emulsion sample was drawn with a pipette and placed on the plate–plate fixture of a rheometer (RS6000, Thermo Fisher Scientific Co., Ltd., Karlsruhe, Germany). A P35TiL rotor was then mounted to measure the static rheological properties of the sample. At 25 °C, the shear rate of the rheometer was set to increase from 0.1 to 100 s^−1^ [14]. The changes in shear stress and apparent viscosity of the emulsion with shear rate were measured, and the shear stress–shear rate curve and apparent viscosity–shear rate curve were obtained.

The power law rheological model was adopted for regression analysis and data fitting, with the goodness of fit assessed via the multiple correlation coefficient (R^2^).

The power law is expressed as: τ = K(γ)^n^

In this formula, τ indicates shear stress (in Pa); K denotes the consistency coefficient (in mPa·s^n^); γ represents the shear rate (in s^−1^); and n stands for the flow behavior index.

### 2.6. Determination of Dynamic Rheological Properties

At 25 °C, a rheometer (RS6000, Thermo Fisher Scientific Co., Ltd.) equipped with the same P35TiL plate–plate geometry as used in steady state measurements was employed. Exactly 1 mL of freshly prepared emulsion sample was transferred onto the test plate, and the plate gap was adjusted to 1 mm. A frequency sweep range of 0.1~10 Hz was set, and the dynamic variation patterns of storage modulus (G′) and loss modulus (G″) with frequency were determined and recorded [15].

### 2.7. Determination of Emulsion Stability Index (ESI)

The determination of ESI was modified with method reported by [16]. A 500 µL aliquot of the emulsion sample was added to 1% SDS solution to make a total volume of 10 mL. The absorbance of the sample was measured at 500 nm at 0 min and 30 min, respectively, to calculate the ESI for evaluating the emulsion stability. The calculation formula of ESI is as follows:

$$\mathrm{ESI}\;(\%)\;=\;100\;\times \;\frac{A_{t}}{A_{0}}$$

where A_0_ and A_t_ are the absorbance values of the emulsion at 0 min and 30 min, respectively.

### 2.8. Determination of Thermal Stability of Emulsions

The emulsions were heated in water baths at 25 °C, 60 °C and 80 °C for 2 h, respectively. The stratification phenomenon of the emulsions after heating was observed and recorded by photography, and the emulsification index (EI) was calculated to evaluate the thermal stability of the emulsions. The calculation formula of EI is as follows:

$$\mathrm{EI}\;(\%)\;=\;\frac{\mathrm{height}\;\mathrm{of}\;\mathrm{emulsified}\;\mathrm{phase}\;(\mathrm{mm})}{\mathrm{total}\;\mathrm{height}\;\mathrm{of}\;\mathrm{oil}/\mathrm{water}\;\mathrm{mixture}\;(\mathrm{mm})}\;\times \;100$$

### 2.9. Observation of Micro-Morphology

A drop of emulsion was placed on a glass slide, covered with a coverslip, and slides of different emulsion samples were prepared. The morphological characteristics of the emulsions were observed under an optical microscope (IX71, Olympus, Tokyo, Japan) at 40× magnification, and images were captured for documentation.

### 2.10. Low-Field Nuclear Magnetic Resonance (LF-NMR) Measurement

10 mL of the emulsion was poured into an LF-NMR (NMI20-015V-I, NIUMAG, Suzhou, China) tube, and the transverse relaxation time was determined using the Carr–Purcell–Meiboom–Gill (CPMG) pulse sequence. The main measurement parameters were as follows: echo time (TE) = 0.400 ms, number of sampling points per echo = 16,384, number of acquired echoes = 12, total number of sampling points (TD) = 199,978, number of scans (NS) = 8, and maximum number of echoes supported by the instrument under these parameters = 20.

The SE sequence was used for imaging the emulsion in the LF-NMR tube, with specific parameters: FOV Phase = 80 mm, FOV Read = 80 mm, echo time (TE) = 20 ms, repetition time (TR) = 500 ms, Slices = 1, Averages = 2, and Slices Width = 2.5 mm [17].

### 2.11. Atomic Force Microscopy (AFM) Observation

The morphology of the emulsion was observed using an atomic force microscope (Bruker Corporation, Billerica, MA, USA). The emulsion sample was diluted to 2 μg/mL with distilled water, and the diluted sample was then dropped onto a mica sheet. Imaging was performed after air-drying. A silicon nitride probe with a tip curvature radius less than 10 nm was used [18].

### 2.12. Statistical Analysis

All experiments were performed in triplicate, and the results were expressed as mean ± standard deviation. The significance of differences was determined by one-way analysis of variance (ANOVA) combined with Duncan’s test (*p* < 0.05). Statistical analyses were conducted using SPSS 26.0 (IBM Corp., Armonk, NY, USA).

## 3. Results and Discussion

### 3.1. Analysis of Reverse Compression Test

**Figure 1:**
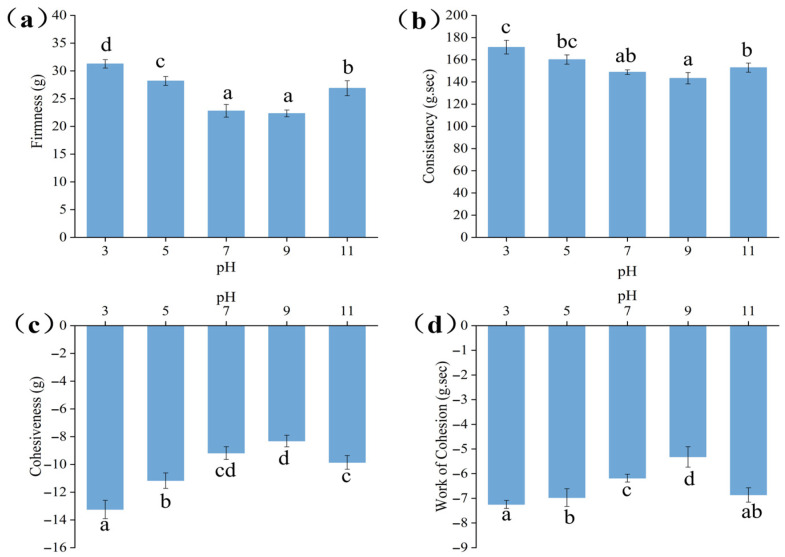
Parameters of reverse compression test characteristics for Pickering emulsions stabilized by the GES-QP complex prepared under different pH conditions (pH 3, pH 5, pH 7, pH 9, pH 11): firmness (**a**), consistency (**b**), cohesiveness (**c**), work of cohesion (**d**). Means annotated with different letters within the same column are significantly different (*p* < 0.05).

Texture properties are one of the core indicators for evaluating the macroscopic application performance of emulsions, directly related to their stability and sensory experience during food processing, storage, and consumption. As a classic structural analysis method, the reverse compression test can intuitively reflect the internal structural strength, droplet dispersion state, and intermolecular interaction intensity of emulsions by quantifying key parameters such as hardness, consistency, and cohesiveness. The results of the reverse compression test on Pickering emulsions stabilized by the GES-QP complex under different pH conditions are presented in Figure 1. The pH value significantly affected the textural properties of the emulsion by regulating the intermolecular forces and interfacial behaviors between GES and QP [19]. At pH 3, the hardness of the emulsion (Figure 1a) reached the maximum value (31.25 ± 0.76 g), followed by a gradual decrease as the pH increased to 9, with a slight rebound to 26.87 ± 1.34 g at pH 11. The isoelectric point of QP is approximately 4.5; thus, in the acidic environment of pH 3, its molecules carried a large number of positive charges [20]. Protonation led to the formation of more hydrogen bonds within the protein molecules, for instance, some groups that originally did not participate in hydrogen bond formation might engage in such interactions after protonation. This enhanced intramolecular interactions, causing the protein molecular chains to contract tightly and form a relatively rigid structure [21]. GES molecules, rich in hydroxyl groups, formed abundant hydrogen bonds with QP molecules. The two tightly bound at the interface, constructing a high-strength interfacial film that effectively resisted external deformation, resulting in high hardness [22]. When the pH deviated from the isoelectric point (e.g., pH 5, 7, 9), QP molecules gradually acquired negative charges, their chains stretched, the hydrogen bond interaction with GES weakened, intermolecular binding force decreased, the supporting capacity of the interfacial film declined, and thus hardness reduced accordingly. Under the strongly alkaline condition of pH 11, QP molecules partially unfolded, exposing more hydrophobic sites, which could bind to GES through hydrophobic interactions. Meanwhile, the flexibility change in GES molecular chains in the alkaline environment restored the interaction between the two, leading to a slight rebound in hardness. The variation law of hardness is directly related to the compactness of the three-dimensional network structure of the emulsion, and a higher hardness value corresponds to a more stable structural support capacity, which is consistent with the “dense protrusion” structural characteristic of the emulsion interface film at pH 3 in the subsequent microscopic analysis.

As a key parameter reflecting the flow resistance and structural integrity of the emulsion, the change trend of consistency is related to the extrusion molding potential of the emulsion. The change in consistency (Figure 1b) was correlated with hardness [23]. The emulsion exhibited the highest consistency at pH 3 (171.25 ± 6.16 g·s), which first decreased and then increased in the pH range of 5–11. At pH 3, positively charged and structurally rigid QP tightly combined with GES via hydrogen bonds, promoting the emulsion to form a continuous and dense three-dimensional network structure [24]. The droplets were arranged closely with small gaps, and reverse compression required overcoming greater internal friction and structural resistance, resulting in the highest consistency. As the pH increased to neutral and weakly alkaline, the charge state of QP molecules changed, their chains stretched, the binding force with GES weakened, the network structure became loose, the distance between droplets increased, the fluidity of the system improved, and thus consistency decreased. At pH 11, the hydrophobic interaction between QP and GES was enhanced. Simultaneously, the alkaline environment strengthened the hydration capacity of GES and increased the degree of molecular chain stretching, but the increase in internal friction resistance of the system was limited [25]. Hence, consistency rebounded but remained lower than that under acidic conditions.

Cohesiveness (Figure 1c) reflects the aggregation ability of droplets within the emulsion [26]. At pH 3, cohesiveness was the weakest (−13.25 ± 0.66 g), indicating low attraction and good dispersibility between droplets. In the acidic environment, since the pH was lower than the isoelectric point of QP, its molecular chains contracted to form a rigid structure, creating effective steric hindrance at the droplet interface. GES molecular chains could fill the gaps between protein molecules, and the two synergistically constructed a strong steric barrier to prevent droplet aggregation. Additionally, the tightly bound GES and QP endowed the interfacial film with high strength, making droplets less prone to deformation and aggregation, which corresponded to the low aggregation characteristics observed in the back-extrusion test. As the pH increased, QP molecular chains stretched, the steric hindrance effect weakened, the binding force between droplets enhanced, and the absolute value of cohesiveness decreased. The change trend of cohesive work was similar to that of cohesiveness. The absolute value of cohesive work (Figure 1d) was the largest at pH 3 (−7.23 ± 0.16 g·s), confirming that under this condition, due to the strong steric barrier and high interfacial film strength between droplets, the binding energy was the lowest. The system was characterized by dispersion stability, which collectively ensured high stability of the emulsion. The results of cohesiveness and work of cohesion mutually confirm that low cohesiveness means the emulsion is not prone to droplet aggregation under force, which is of great significance for maintaining the uniformity of the emulsion during storage.

### 3.2. Analysis of Static Rheological Properties

**Figure 2:**
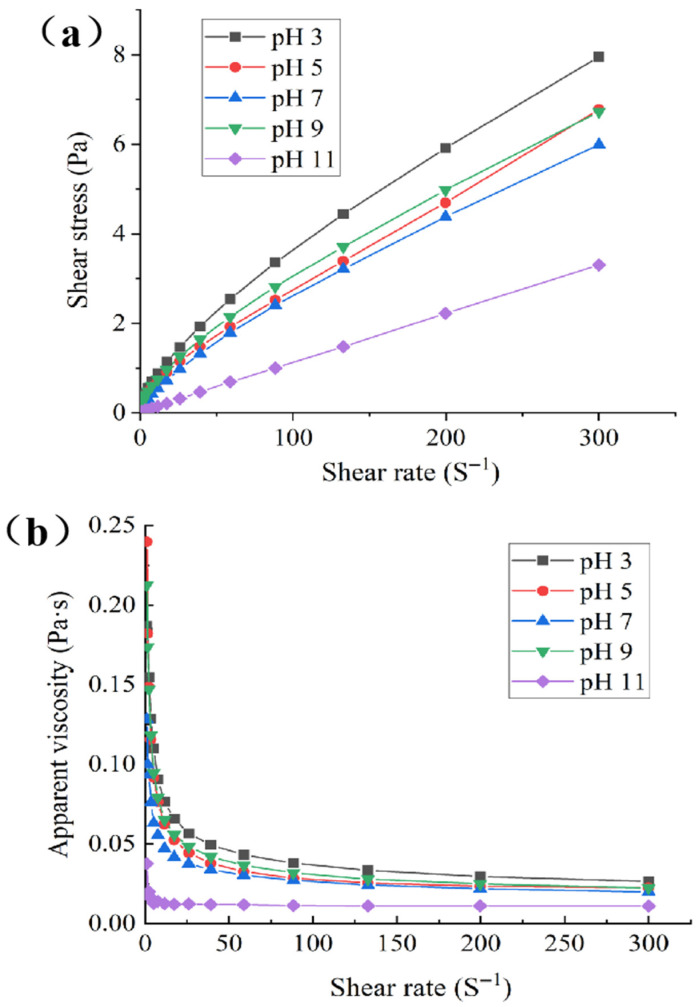
Shear stress versus shear rate (**a**) and apparent viscosity versus shear rate (**b**) for emulsions stabilized by the GES-QP complex prepared under different pH conditions (pH 3, pH 5, pH 7, pH 9, pH 11).

Figure 2 presents the static rheological test results of Pickering emulsions stabilized by the GES-QP complex under different pH conditions, and Table 1 shows the parameter results obtained by fitting the test data with the power–law model. The two mutually verified the regulatory effect of pH on the rheological properties of emulsions from the perspectives of experimental phenomena and quantitative indicators [27]. As analyzed from the shear stress–shear rate curve (Figure 2a), the shear stress of all emulsions increased with the increase in shear rate. This phenomenon indicated that the internal structure of the emulsion could transmit stress through interactions when subjected to shear. Consistent with the high fitting degree (R^2^ ≥ 0.995) of all systems in the power–law model fitting results (Table 1), this confirmed that the flow behavior of the emulsions conformed to the characteristics of the power–law model. At pH 3, QP and GES formed a dense network, leading to the fastest increase and highest value of shear stress. At pH 5, 7, and 9, the aggregation degree of protein and starch was relatively weak, so the shear stress increased moderately with similar trends among the three groups. At pH 11, the strong alkaline environment destroyed the starch molecular structure, resulting in a weak system network, and thus the shear stress increased slowly with the lowest value.

Figure 2b shows the change in apparent viscosity of the emulsions with shear rate. All emulsions exhibited shear-thinning behavior, i.e., the apparent viscosity decreased with the increase in shear rate, reflecting that shear disrupted the internal structure of the emulsions [28]. This was consistent with the quantitative result of flow index *n* < 1 for all systems in Table 1. At pH 3, the high molecular friction caused by protein aggregation resulted in the highest viscosity; this high internal friction originated from the compact and stable network structure, which could effectively hinder droplet coalescence and facilitate emulsion stability. The emulsion systems at pH 5, 7, and 9 had loose structures and low initial viscosities. With the increase in shear rate, the internal network structure of the emulsions was destroyed, leading to a decrease in viscosity. At pH 3, the strong interaction between protein and starch maintained a relatively high final viscosity; the certain structural strength retained during shearing helped to maintain emulsion stability. At pH 11, the strong alkaline environment destroyed the macromolecular structure, and the viscosity rapidly decreased to the lowest level and stabilized. The high initial viscosity and retained structural strength at pH 3 enabled the emulsion to effectively resist external disturbances such as shear.

As shown in Table 1, the system at pH 3 formed a dense network, characterized by the highest consistency coefficient (K) and the smallest *n*, exhibiting the most significant shear-thinning property and strong viscosity retention capacity. At pH 5, 7, and 9, there were slight differences in the looseness of the system network, with K and n values in the middle range and moderate shear-thinning degree. At pH 11, the strong alkaline environment destroyed the macromolecular structure, resulting in the lowest K value and the *n* value closest to 1; the flow pattern approached that of a Newtonian fluid, and the viscosity rapidly decreased to the lowest level.

### 3.3. Analysis of Dynamic Rheological Properties

**Figure 3:**
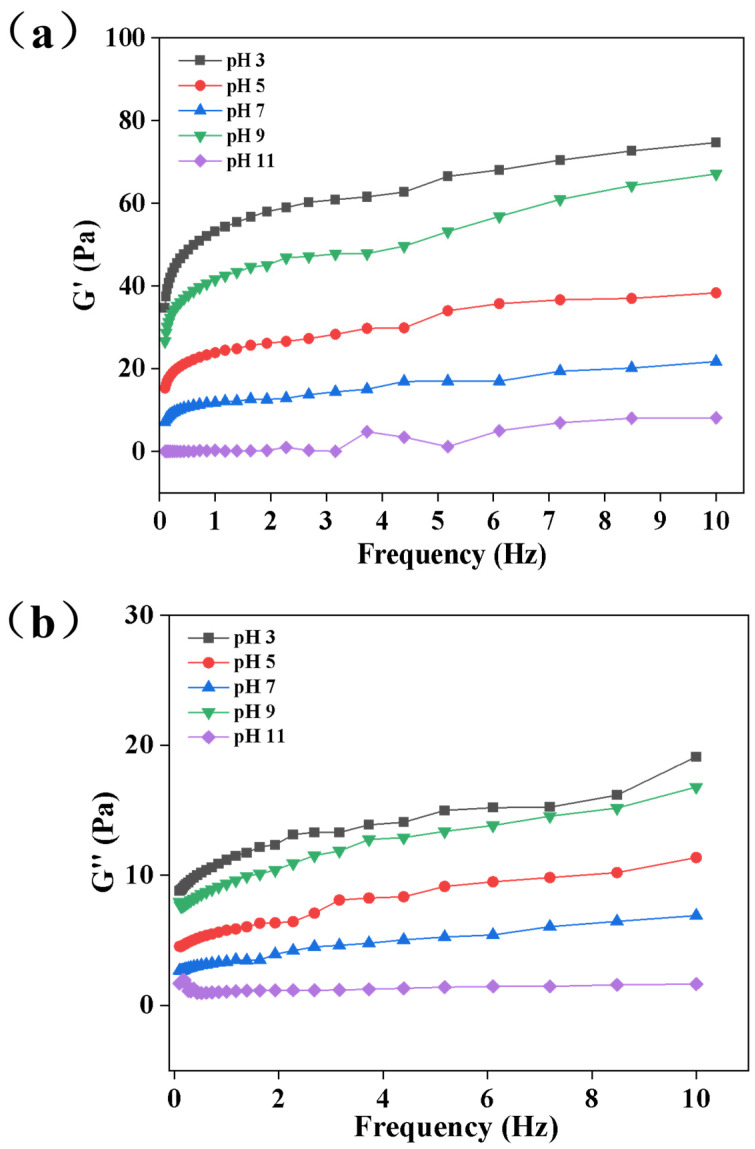
Storage modulus (G′) versus frequency (**a**) and loss modulus (G″) versus frequency (**b**) for emulsions stabilized by the GES-QP complex prepared under different pH conditions (pH 3, pH 5, pH 7, pH 9, pH 11).

Dynamic rheological tests were conducted to investigate the viscoelastic characteristics of emulsions, reflecting the strength and stability of the internal network structure, with the results shown in Figure 3. Storage modulus (G′) characterizes the elastic behavior of emulsions, reflecting the rigidity of the network structure, while loss modulus (G″) represents the viscous behavior, indicating the fluidity of the system. Under all pH conditions, the G′ values of emulsions were significantly higher than G″, and both increased with increasing frequency, demonstrating that the emulsions were dominated by elasticity, exhibited typical gel-like properties, and possessed a stable three-dimensional network structure internally. Among them, the pH 3 group showed the highest G′ and G″ values with the most significant increment as frequency increased, indicating that under this condition, Euryale ferox starch and quinoa protein formed a dense and rigid network structure through strong hydrogen bonding interactions, endowing the emulsion with excellent structural support capacity. The G′ and G″ values of the pH 5, 7, and 9 groups all decreased, suggesting that the change in the charged state of quinoa protein led to the unfolding of molecular chains, weakened hydrogen bonding interactions with starch, resulting in a loose network structure and reduced elasticity and viscosity. The pH 11 group exhibited the lowest G′ and G″ values, which was attributed to the partial hydrolysis of glycosidic bonds in both protein and starch under the strong alkaline environment, leading to insufficient structural strength.

### 3.4. Analysis of ESI

**Figure 4:**
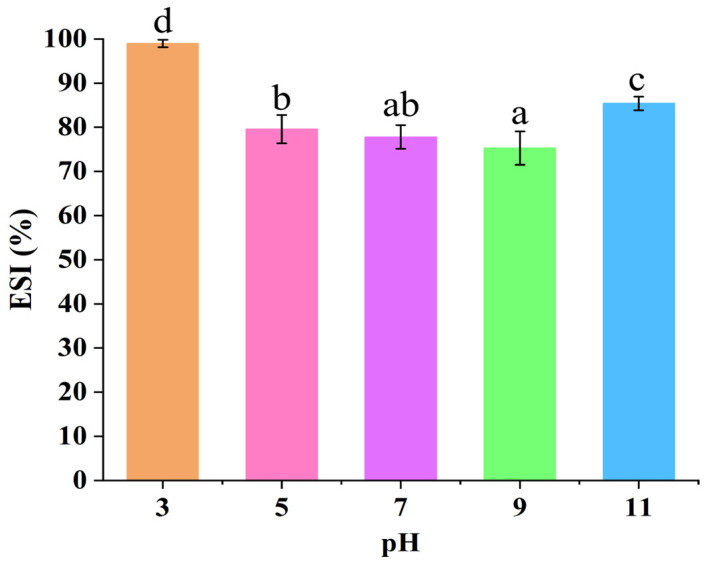
ESI of Pickering emulsions stabilized by the GES-QP complex prepared under different pH conditions (pH 3, pH 5, pH 7, pH 9, pH 11). Means annotated with different letters within the same column are significantly different (*p* < 0.05).

Different pH conditions significantly affected the ESI of Pickering emulsions prepared using the GES-QP complex (Figure 4). Since the isoelectric point of QP is approximately 4.5, its molecules contain dissociable groups such as amino and carboxyl groups. In contrast, GES is composed of glucose residues linked by glycosidic bonds and contains a large number of hydroxyl groups with no inherent charge. At pH 3, as the environmental pH was lower than the isoelectric point of QP, QP molecules carried a large number of positive charges. Their rigid molecular chains tightly combined with the hydroxyl groups of GES through hydrogen bonds and van der Waals forces, synergistically constructing an interfacial film with relatively high strength at the oil–water interface. This strong interfacial film effectively hindered droplet coalescence, endowing the emulsion with good emulsifying stability and thus a higher ESI [29]. When the pH shifted toward neutrality (pH 5, 7) and alkalinity (pH 9, 11), the charge property of QP molecules changed with an increase in negative charges, and the molecular chains gradually stretched from a contracted state. This weakened the hydrogen bond interaction with GES, reduced the strength of the interfacial film, and made droplets prone to coalescence upon collision, leading to a subsequent decrease in ESI. Under the strongly alkaline environment of pH 11, QP molecules partially unfolded, exposing more hydrophobic sites, which enhanced the hydrophobic interaction with GES and repaired the intermolecular binding force to a certain extent, resulting in a slight rebound in ESI. However, due to the potential damage of alkalinity to the molecular structures of protein and starch (e.g., partial hydrolysis of starch glycosidic bonds), the stability did not recover to the level observed at pH 3.

### 3.5. Analysis of Emulsion Thermal Stability

**Figure 5:**
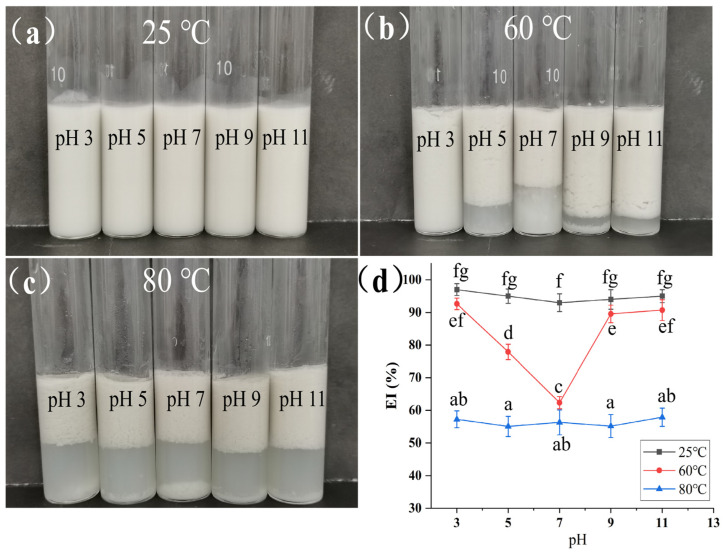
Appearance photographs of Pickering emulsions stabilized by the GES-QP complex prepared under different pH conditions (pH 3, pH 5, pH 7, pH 9, pH 11) after heating at 25 °C (**a**), 60 °C (**b**), and 80 °C (**c**), respectively, and EI (**d**) of the above emulsions after heating. Means annotated with different letters within the same column are significantly different (*p* < 0.05).

The observations of emulsion stratification and changes in EI after heating the emulsions prepared under different pH conditions at various temperatures are shown in Figure 5. At 25 °C, all emulsions in each pH group appeared as uniform white emulsions (Figure 5a), since the temperature did not damage the interfacial film and network structure constructed by starch and protein, and the droplets remained stably dispersed. After heating at 60 °C for 2 h (Figure 5b), the emulsion at pH 3 still maintained a uniform emulsion state, showing strong thermal stability. the pH 5, 7, and 9 groups exhibited obvious stratification. Due to the weak binding force between starch and protein, high temperature intensified droplet movement and collision, and the interfacial film failed to resist coalescence, resulting in oil phase separation [30], the emulsion at pH 11 showed a small amount of precipitation, but the degree of stratification was weaker than that of the pH 5, 7, and 9 groups because hydrophobic interactions repaired part of the binding force. At a high temperature of 80 °C (Figure 5c), the high-temperature environment further intensified the unfolding of protein molecular chains and the partial hydrolysis of starch glycosidic bonds. As a result, the interfacial film completely lost its mechanical support, and the droplets of all emulsion samples aggregated rapidly and underwent oil-phase separation.

At 25 °C, the EI of each pH group (Figure 5d) showed a similar trend to ESI, and the emulsion at pH 3 maintained a high EI value due to the stable interfacial film. After heating at 60 °C, the EI of the emulsion at pH 3 decreased slightly. At 80 °C, the EI of all emulsions decreased significantly.

### 3.6. Analysis of Microscopic Morphological Characteristics

**Figure 6:**
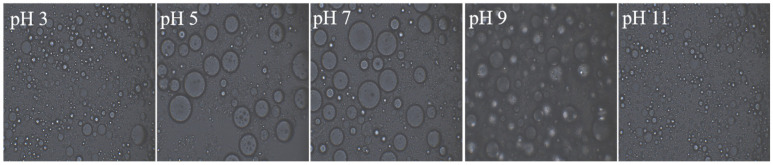
Optical microscopy images of Pickering emulsions stabilized by the GES-QP complex prepared under different pH conditions (pH 3, pH 5, pH 7, pH 9, pH 11).

Under different pH conditions, the morphologies of Pickering emulsions stabilized by the GES-QP complex showed significant differences (Figure 6). At pH 3, the emulsion droplets were relatively fine and dense. This was because QP carried positive charges at pH 3, with its molecular chains contracting into a rigid state, and tightly combined with GES through hydrogen bonds, synergistically forming a dense interfacial film at the oil–water interface. As a result, the emulsion droplets had small particle sizes and relatively uniform distribution [31]. With the increase in pH (pH 5, 7, 9), the particle sizes of emulsion droplets began to increase and the distribution became uneven. This was attributed to the change in the charge property of QP molecules (weak positive charges at pH 5, negative charges at pH 7 and 9), which caused the molecular chains to stretch, weakened the hydrogen bond interaction with GES, reduced the stability of the interfacial film, and made the droplets prone to coalescence and growth. Under the strongly alkaline environment of pH 11, QP was partially unfolded, and the hydrophobic interaction with GES was enhanced, which inhibited excessive coalescence of droplets to a certain extent. Thus, the droplet morphology was finer and denser compared with that of the pH 5, 7, and 9 groups.

### 3.7. LF-NMR Imaging and Moisture Distribution Analysis

**Figure 7:**
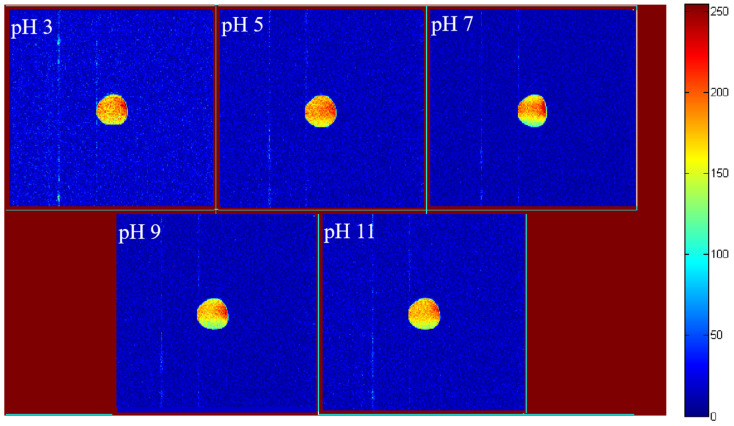
LF-NMR images of Pickering emulsions stabilized by the GES-QP complex prepared under different pH conditions (pH 3, pH 5, pH 7, pH 9, pH 11).

The pseudo-color images of LF-NMR moisture distribution (Figure 7) can intuitively reflect the moisture state in the emulsion. At pH 3, the moisture pseudo-color image of the emulsion showed uniform and high-intensity signals (bright colors). This was because the strong interfacial film and dense three-dimensional network constructed by GES and QP had a strong ability to bind moisture, and the moisture stably existed in the system in the form of bound water and weakly bound water [32]. In the pH 5, 7, and 9 groups, as the interaction between GES and QP weakened, the interfacial film and network structure became loose, the moisture-binding capacity decreased, the proportion of free water increased, and the signal intensity of the pseudo-color images decreased with dispersed distribution (dim colors and wide range). At pH 11, hydrophobic interactions helped restore part of the intermolecular binding force, so the moisture-binding capacity was improved, and the signal intensity of the pseudo-color image was higher than that of the pH 5, 7, and 9 groups. However, due to the structural damage of starch and protein molecules in the alkaline environment, the moisture distribution was still not as uniform and stable as that of the emulsion at pH 3, which reflected the law that pH affects the moisture occurrence state by regulating molecular interactions.

**Figure 8:**
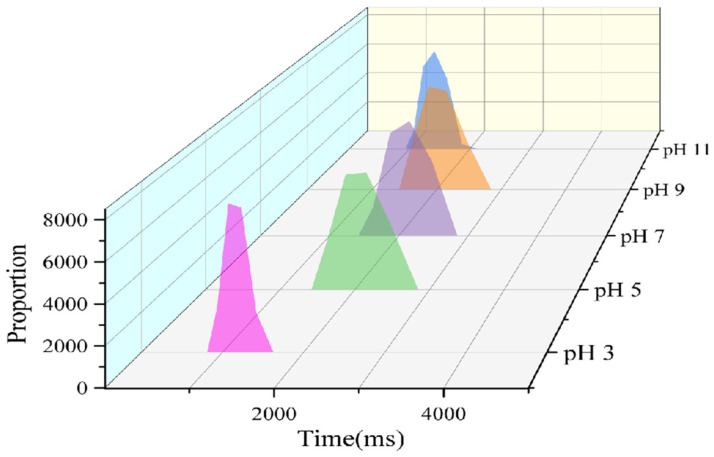
Relaxation time distributions of Pickering emulsions stabilized by the GES-QP complex prepared under different pH conditions (pH 3, pH 5, pH 7, pH 9, pH 11).

The moisture proton relaxation time (T2) (Figure 8) reflects the fluidity and binding state of moisture in the emulsion, and its distribution characteristics can be directly linked to the emulsion stability, droplet coalescence, and thermal separation behavior of the emulsion, providing quantitative support for the mechanism of system stability [33]. At pH 3, the relaxation time was short and the distribution was concentrated (the peak for pH 3 in Figure 8 was narrow and shifted to the left), indicating that moisture was strongly bound by the interfacial film and network structure, with extremely low fluidity, mainly in the form of tightly bound water, which corresponded to the high stability of the emulsion. This strong water-binding effect can, on the one hand, reduce the probability of droplet collision mediated by free water and effectively inhibit droplet coalescence; on the other hand, it can decrease the water migration rate, delay emulsion creaming, enhance the structural integrity of the interfacial film, improve the emulsion’s tolerance to high temperatures, and reduce the risk of thermal separation. Ultimately, this corresponds to the relatively high emulsification stability of the emulsion [34]. In the pH 5, 7, and 9 groups, the relaxation time was prolonged and the peak width increased (the peaks for pH 5, 7, and 9 in Figure 8 were wide and shifted to the right). Due to the decrease in intermolecular binding force, the binding of the interfacial film and network structure to moisture was weakened, the proportion of free water increased, and moisture fluidity was enhanced, making droplets prone to collision and coalescence mediated by moisture, thus reducing emulsion stability. For the emulsion at pH 11, the moisture-binding capacity was improved, and the relaxation time was shorter and the peak shape was narrower compared with those of the pH 5, 7, and 9 groups. This further confirmed that pH changed moisture binding and fluidity by regulating the interaction between GES and QP, ultimately affecting the stability of the emulsion. The quantitative differences in T2 distribution further confirmed that pH affected the stability of the emulsion by regulating the network compactness and water occurrence state of the GES-QP composite system, among which a single narrow T2 peak with a relatively short peak value could help improve the emulsion stability.

### 3.8. AFM Morphology Analysis

**Figure 9:**
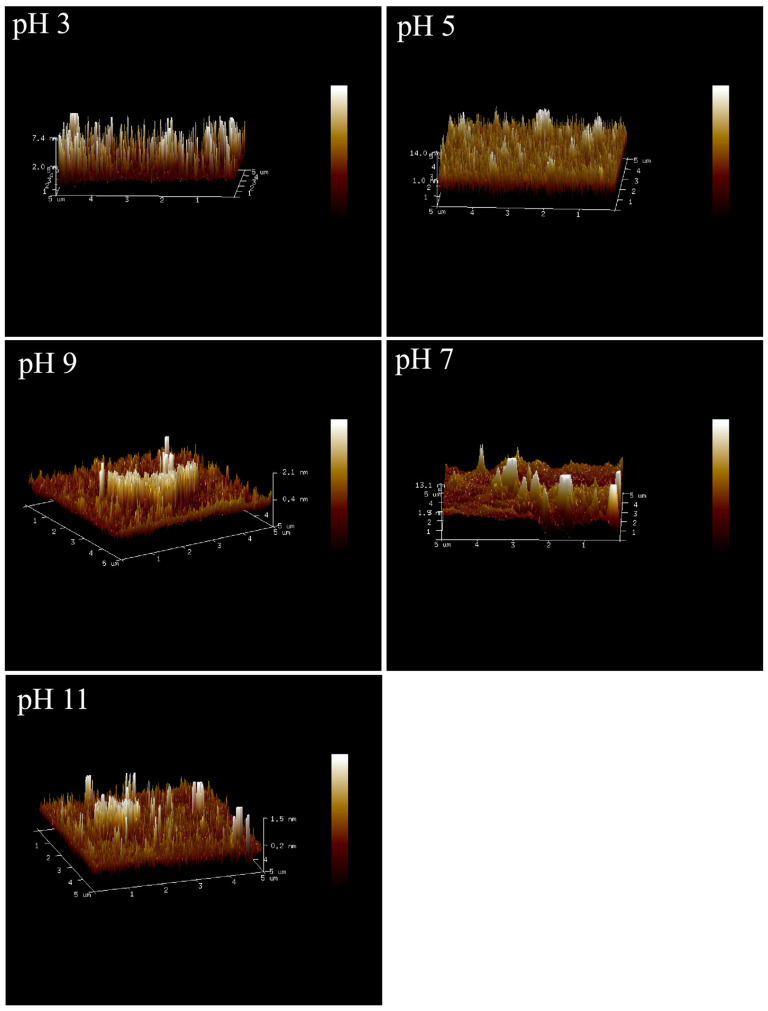
AFM 3D image profiles of Pickering emulsions stabilized by the GES-QP complex prepared under different pH conditions (pH 3, pH 5, pH 7, pH 9, pH 11).

Under different pH conditions, the three-dimensional (3D) morphologies of Pickering emulsions observed by AFM (Figure 9) showed significant differences, reflecting the influence of pH on the microstructure of the interfacial film by regulating intermolecular interactions. At pH 3, the surface of the emulsion sample exhibited dense peaks and ridges. The lateral diameter was approximately 0.1~1.0 μm, and the height was about 1.0~7.4 nm. The interaction between GES and QP promoted the formation of a “dense protrusion” structure in the interfacial film, where rigid protein chains and flexible starch chains synergistically constructed a “rigid–flexible integrated” interface. This structure could provide high mechanical support for the emulsion, inhibit droplet coalescence, and improve stability [35]. With the increase in pH (pH 5, 7, 9), the 3D morphologies of the emulsions showed phenomena such as reduced peak height, uneven distribution, and flat surface. The lateral diameter increased to 0.5~3.0 μm, with a height of around 2.0~13.1 nm. This was because the charge property of QP molecules gradually changed, and the molecular chains stretched gradually from a contracted state, leading to the attenuation of hydrogen bond interactions with GES. The interfacial film began to weaken due to the reduced synergistic effect between starch and protein, and its microstructure tended to be smooth with decreased mechanical strength, making it difficult to effectively resist droplet collisions. As a result, droplets were prone to coalescence, and emulsion stability decreased. Under the strongly alkaline environment of pH 11, the micro-protrusions of the interfacial film were partially restored and distributed relatively uniformly. The lateral diameter was roughly 0.3~2.0 μm, and the height was approximately 1.0~1.5 nm. The differences in the microstructure of emulsions observed by atomic force microscopy could directly confirm the macroscopic stability of the emulsions (e.g., emulsifying stability index, thermal stability, etc.).

## 4. Conclusions

This study investigated the structure–activity relationship and stability mechanism of Pickering emulsions stabilized by the GES-QP complex under different pH conditions (3, 5, 7, 9, 11). At pH 3, the emulsion exhibited the highest hardness and consistency, along with the weakest cohesiveness. At pH 11, the textural properties slightly recovered. All emulsions showed shear-thinning behavior. pH 3 resulted in the highest shear stress and apparent viscosity with a stable system network, while pH 11 led to the lowest viscosity due to macromolecular structure damage. The emulsion at pH 3 demonstrated the optimal stability, maintaining a homogeneous state after heating at 60 °C; stability significantly decreased at pH 5–9 due to weakened hydrogen bonding; at pH 11, hydrophobic interactions partially restored stability but it remained weaker than that at pH 3. Microscopic analyses (optical microscopy, LF-NMR, and AFM) further confirmed that at pH 3, the emulsion droplets were fine and uniform, with strong water-binding capacity and a “dense protrusion” interfacial film structure; at pH 5–9, droplet coalescence occurred, water mobility increased, and the interfacial film structure became flat; at pH 11, the droplet morphology and interfacial structure slightly improved. pH can regulate the interactions between GES and QP, altering the charge state of composite particles, interfacial film structure, and water occurrence state, thereby influencing the macroscopic properties and stability of the emulsion. This study provides experimental basis for designing functional emulsions with strong environmental adaptability and lays a theoretical foundation for the high-value utilization of gorgon euryale and quinoa resources, and can also serve as a safe and stable natural carrier for the encapsulation and delivery of fat-soluble active ingredients in the food, pharmaceutical, and cosmetic fields.

## Figures and Tables

**Table 1:** The parameters of power–law model of the emulsions stabilized by the GES-QP complex prepared under different pH conditions (pH 3, pH 5, pH 7, pH 9, pH 11). Means annotated with different letters within the same column are significantly different (*p* < 0.05).

Samples	Parameters of Power–Law Model		
	K (mPa·s^n^)	*n*	R^2^
GES-QP (pH 3)	158.13 ± 1.26 ^e^	0.68 ± 0.02 ^a^	0.999 ± 0.001 ^a^
GES-QP (pH 5)	99.62 ± 0.38 ^c^	0.73 ± 0.01 ^b^	0.995 ± 0.002 ^a^
GES-QP (pH 7)	88.66 ± 0.70 ^b^	0.74 ± 0.04 ^b^	0.999 ± 0.001 ^a^
GES-QP (pH 9)	136.09 ± 0.83 ^d^	0.68 ± 0.03 ^a^	0.997 ± 0.002 ^a^
GES-QP (pH 11)	13.37 ± 0.14 ^a^	0.96 ± 0.02 ^c^	0.999 ± 0.001 ^a^

## Data Availability

The original contributions presented in this study are included in the article. Further inquiries can be directed to the corresponding authors.
